# Employees’ Organizational Identification and Affective Organizational Commitment: An Integrative Approach

**DOI:** 10.1371/journal.pone.0123955

**Published:** 2015-04-13

**Authors:** Florence Stinglhamber, Géraldine Marique, Gaëtane Caesens, Donatienne Desmette, Isabelle Hansez, Dorothée Hanin, Françoise Bertrand

**Affiliations:** 1 Department of Psychology, Université catholique de Louvain, Louvain-la-Neuve, Belgium; 2 Department of Psychology, Université de Liège, Liège, Belgium; 3 DGHR-Section Recrutement et Sélection-Research & Development, Belgian Defense, Brussels, Belgium; Universiteit Utrecht, NETHERLANDS

## Abstract

Although several studies have empirically supported the distinction between organizational identification (OI) and affective commitment (AC), there is still disagreement regarding how they are related. Precisely, little attention has been given to the direction of causality between these two constructs and as to why they have common antecedents and outcomes. This research was designed to fill these gaps. Using a cross-lagged panel design with two measurement times, Study 1 examined the directionality of the relationship between OI and AC, and showed that OI is positively related to temporal change in AC, confirming the antecedence of OI on AC. Using a cross-sectional design, Study 2 investigated the mediating role of OI in the relationship between three work experiences (i.e., perceived organizational support, leader-member exchange, and job autonomy) and AC, and found that OI partially mediates the influence of work experiences on AC. Finally, Study 3 examined longitudinally how OI and AC combine in the prediction of actual turnover, and showed that AC totally mediates the relationship between OI and turnover. Overall, these findings suggest that favorable work experiences operate via OI to increase employees' AC that, in turn, decreases employee turnover.

## Introduction

Over the past decades, a growing body of research has been dedicated to the relationship between employees and their employing organization. Two main perspectives have received increasing attention from scholars. On the one hand, this psychological relationship has been conceptualized in terms of organizational identification (OI). Stemming from the social identity perspective [1,2], OI is defined as "the perception of oneness with or belongingness to an organization, where the individual defines him or herself in terms of the organization(s) in which he or she is a member" [3 p104]. The social identity perspective holds that individuals classify themselves and others into different social categories in order to define and locate themselves in their social environment. In line with this assumption, several scholars have argued that the organization is one of the most relevant foci of identification for the individual (e.g. [4,5]).

On the other hand, the psychological relationship between employees and their employer has also been conceptualized in terms of organizational commitment. Meyer and Herscovitch defined commitment as "a force that binds an individual to a course of action of relevance to one or more targets" [6 p301]. As a multidimensional construct, organizational commitment was considered as encompassing three dimensions [7]. Affective commitment (AC) refers to "an emotional attachment to, identification with, and involvement in the organization" [7 p67]. Normative commitment is viewed as "a feeling of obligation to continue employment" [7 p67]. Finally, continuance commitment is defined as an "awareness of the costs associated with leaving the organization" [7 p67].

Despite the evident similarities between OI and organizational commitment (and, in particular, AC), these perspectives have largely developed independently from each other. Scholars who have recently brought together these two fields of research (e.g. [8]) have thus begun to question the discriminant validity of OI and AC. So far, the distinction between these two constructs has been supported at both the theoretical (e.g. [9,10]) and the empirical level (e.g. [11]). There is thus relative consensus amongst scholars on the distinctiveness of these two concepts.

Although distinguishable, OI and AC have been found to be strongly related. Many studies have reported a positive relationship between the two constructs [12]. Despite the attention OI and AC have received, there is still considerable disagreement regarding how they are related [8]. First, little attention has been given to assessing the direction of causality between these two constructs and the potential role of one concept in the development of the other. Second, insufficient consideration has been given as to why OI and AC have common antecedents and outcomes. Filling these important gaps, we argue in this research that OI (i.e. a cognition-based construct) is a determinant of AC (i.e. an affect-based concept), whereas the reverse cannot be stated. In agreement with this proposition, we suggest that OI and AC are part of a causal chain where their common antecedents (here, perceived organizational support, leader-member exchange, and job autonomy) predict OI which, in turn, influences AC, which is the proximal determinant of their common outcomes (here, actual turnover). The present research contributes to a broader view of employee-employer relationships that incorporates both literature on organizational identification and literature on affective organizational commitment. By revealing that both constructs are necessary for a complete understanding of these relationships, it shows that critical progress in this domain is best served by an integrative approach.

### The Directionality of the Relationship Between OI and AC

A variety of theoretical perspectives has been proposed on how OI and AC are related. Some authors have suggested that AC is part of the OI concept (e.g. [13]), whereas others have considered OI as a part of the AC construct (e.g. [14]). Finally, some scholars have argued that OI is an antecedent of AC (e.g. [15]). This latter view stems from the common conceptualization of OI as a cognitive construct referring to the self-definitional component of identification [9]. Self-defining as an organizational member might be a precursor to developing positive attitudes toward this organization such as an emotional attachment to this organization. This view was theoretically supported by several scholars in the literature. For instance, Ashforth and Mael argued that identification can "enhance support for and commitment to the organization" [9 p26]. In a similar vein, Meyer et al. [15] suggested that OI fosters AC toward the organization. Similarly, Becker and his colleagues [8,16] proposed that identification with a group often involves the adoption of attitudes, including commitment, directed toward this group. Finally, Meyer and Allen argued that “employees want to remain (i.e., affective commitment) and are willing to exert effort on behalf of the organization because of the benefits they derive from the relationship” [7 p76]. To the extent that identification helps employees to maintain a positive self-image [17], OI should benefit employees and, as such, reinforces their AC toward the organization. This perspective was favorably received in the recent literature in organizational psychology, so this is currently the dominant approach at the conceptual level [8].

This view that OI plays a role in AC’s development is also consistent with Tajfel’s work on the minimal group situation (e.g. [18–20]) which indicates that individuals' knowledge of their group membership is the only necessary requirement for the establishment of a relationship with a group. Cognitive awareness of group membership would be the first step in developing this relationship and, albeit not systematic, emotional states or behaviors can subsequently arise from individuals' self-categorization as a group member (e.g. [9,21]). The cognition-affect-behavior sequence can also be found in theoretical and empirical work on intergroup relations. This latter literature has traditionally proceeded on the assumption that stereotyping promotes prejudice which, in turn, dictates discrimination (e.g. [22–24]). In other words, beliefs about what a group is like are assumed to cause liking or disliking for the group which, in turn, leads to favorable or unfavorable actions toward it. This traditional causal sequence between cognition, affect and behavior was heavily influenced by early conceptions of attitudes (e.g. [25]). Evident parallels can be drawn between the conceptualization of stereotypes, prejudice and discrimination and the conceptualization of beliefs, attitudes and behaviors in the literature on attitudes [23].

Empirically, some preliminary evidence supports the proposition that OI is an antecedent of AC. Ellemers and her colleagues [26] found in two experimental studies that group commitment was predicted by in-group identification. Bergami and Bagozzi [4] and Marique and Stinglhamber [27] also found that OI predicted AC, whereas the reverse was not true. Furthermore, Carmeli, Gillat, and Weisberg [28] showed that OI mediated the relationship between organizational prestige and AC. Finally, Marique, Stinglhamber, Desmette, Caesens, and De Zanet [29] found that OI mediated the interactive effect of perceived organizational support and organizational prestige on AC, and that AC mediated the effect of OI on extra-role performance.

However, these field studies are based on cross-sectional data, leaving uncertain the direction of causality in the relationship between OI and AC in real organizational settings. A relationship between the initial value of one variable and changes in a second variable over time provides stronger causal evidence than is afforded by the simultaneous measurement of the two variables [30,31]. While no advanced correlation technique can fully substitute for experimental procedures in providing evidence of causality, cross-lagged panel designs are “typically considered the optimal way to understand causality in field settings where experimental procedures are not feasible” [32]. In the present research, we therefore investigated the causal relationship between OI and AC using such a design. Specifically, we posited that initial OI would be related to temporal change in AC.

*Hypothesis 1*: OI is positively related to temporal change in AC.

### The Mediating Role of OI in the Relationship Between Favorable Work Experiences and AC

AC has been shown to be related to a broad range of variables supposed to be its antecedents [33]. This variety of determinants can be categorized into three specific groups: organizational characteristics, individual characteristics, and work experiences [34]. Among these categories, 'work experiences' is certainly the one that includes the largest number of studies, reporting the strongest and most consistent results. It is simply impossible to enumerate an exhaustive list of antecedents pertaining to this group. However, as pointed out by Meyer and Allen [34], two common psychological themes emerge from this body of research. In particular, work experiences (a) indicating that the organization treats its employees favorably and (b) enhancing employees' sense of personal importance and competence are very likely to induce an emotional attachment to the organization.

In line with the first theme, a number of studies showed that favorable treatment in terms of supportiveness or fairness from the organization or its agents is a key predictor of AC. In this study, we focused on two determinants of AC corresponding to this theme and important in this literature, i.e. perceived organizational support and leader-member exchange [35]. Perceived organizational support is defined as employees' beliefs concerning the extent to which the organization values their contributions and cares about their well-being [36]. Many studies have shown a strong link between perceived organizational support and AC (for a review, see [37]). Importantly, Rhoades and her colleagues [38] have provided evidence through a cross-lagged panel design for the antecedence of perceived organizational support on AC. The relationship between these two concepts has been primarily explained in terms of social exchange (e.g. [39]). More precisely, perceived organizational support increases AC by creating a felt obligation to care about the organization's welfare and to help it to reach its goals [38].

Leader-member exchange captures the quality of the exchange relationship that develops between an employee and his/her supervisor [40]. Research has consistently demonstrated a positive association between leader-member exchange and AC [35,41]. This relationship is explained by the fact that employees view supervisors as organizational agents [42–44]. Employees therefore generalize their view concerning the favorableness of their exchange relationship from supervisor to organization and reciprocate this favorable treatment by being committed to the organization.

In line with the second theme, numerous studies have shown that work experiences (e.g. participation in decision making, job challenge) that make employees feel important for and competent in the organization, enhance their AC [33]. In the present study, we focused on one specific work experience corresponding to this theme and that has largely been studied as an important determinant of AC, i.e. job autonomy [45,46]. Job autonomy reflects the extent to which a job allows the freedom, independence, and discretion necessary to schedule work and to decide what procedures to use in carrying it out [47]. Many studies reported a positive association between job autonomy and AC (e.g. [46]).

Interestingly, these three antecedents of AC have also been found to be related to OI. Several studies reported a positive link between perceived organizational support and OI (e.g. [48–50]). In the same vein, leader-member exchange was found to be positively related to OI (e.g. [50–51]). Finally, several authors found a positive relationship between job autonomy and OI (e.g. [52–53]). However, little attention has been given in the literature as to why OI and AC are related to the same variables hypothesized to be their predictors. In line with the recent research of Marique et al. [29], we argue in the present paper that these common significant relationships may be explained by the mediating role of OI in the relationships between AC and its antecedents.

A core assumption of social identity theory is that people are motivated to achieve and maintain positive concepts of themselves. As OI refers to the extent to which employees define themselves by organizational attributes, employees should thus be prone to develop OI with organizations that are viewed favorably by their members. Yet, providing personnel with favorable work experiences is certainly a way for organizations to enhance a positive perception and image of themselves, thus making OI more likely [54,55]. Furthermore, Meyer and Allen [34] suggested that work experiences increase individuals' AC by fulfilling their need to feel that they are worthwhile persons. With this proposition, they argue that notions of self-worth or self-esteem are at the core of the antecedents-AC relationships. Similarly, in the literature on perceived organizational support, Rhoades and her colleagues argued that "perceived organizational support would also increase affective commitment by fulfilling needs for esteem, approval, and affiliation, leading to the incorporation of organizational membership and role status into social identity" [38 p825]. Supporting this view, Eisenberger and Stinglhamber [37] proposed that the fulfillment of socioemotional needs is a basic mechanism contributing to the effects of perceived organizational support on several outcomes.

In line with the above arguments, we argue that, in meeting socioemotional needs, the favorable work experiences studied in the present research enhance the attractiveness of the organization and, therefore, increase the likelihood of employees' OI (e.g. [37,50]). In agreement with Hypothesis 1, this higher OI should, in turn, lead employees to feel emotionally attached to the target organization.

*Hypothesis 2*: OI mediates the positive relationship between (a) perceived organizational support, (b) leader-member exchange and (c) job autonomy, and AC.

### The Mediating Role of AC in the Relationship Between OI and Employee Turnover

Employees' retention is becoming one of the key issues for many organizations that are striving to retain talented employees in order to be competitive in the today's economic market. Understanding the factors that shape voluntary turnover is primordial given its considerable financial, social and productivity costs (e.g. [56,57]). Following Terry, Smith, Smith, Amiot, and Callan [58], the costs of losing an employee range from 1/2 to 5 times that employee’s annual salary.

Maertz and Griffeth identified eight motivational forces that “initiate engagement in the mental behavior of turnover deliberations and the physical behavior of actually resigning/quitting [59 p669]. One of these forces is based on a hedonistic approach–avoidance mechanism. The authors assumed that people are generally hedonistic, approaching situations that make them feel good and avoiding situations that make them feel bad. As a consequence, employees who feel good about their current organization and enjoy membership are thereby motivated to stay with this employer. On the contrary, employees who feel negative toward the organization will avoid the psychological discomfort associated with working for this employer and are thus more likely to quit. Consistently, they argued that both OI and AC are in this category of antecedents of voluntary turnover. In line with this view, both OI and AC have been found to be related to intended and actual turnover (e.g. [12,33,60]).

However, insufficient consideration has been given as to why OI and AC share this common outcome. Despite pointing out these variables as important predictors of turnover, Maertz and Griffeth [59] did not consider the potentially different role that OI and AC might play in the process. Nevertheless, some scholars suggested that AC may be more closely aligned with or more proximal to turnover than is OI [11,17,61]. First, the desire to stay in the organization is more central to the AC construct than to the OI concept. The seminal definition of AC [7] comprises the desire to maintain organizational membership, so the measurement of commitment partly overlaps with the measurement of turnover intentions. In contrast, maintaining organizational membership is not considered as a core part of the OI concept even though a negative link with turnover is expected since "people may be reluctant to give up the part of their self-definition that is tied to organizational membership" [11 p575]. Furthermore, to the extent that OI refers to the incorporation of the organization in the self-concept while AC reflects an emotional attitude toward the organization, van Knippenberg and Sleebos [11] argued that AC should be more strongly related to work attitudes and behaviors than is OI. As a whole, this theoretical evidence suggests that OI would influence AC which, in turn, predicts turnover.

As mentioned earlier, the proposed sequence between OI (i.e., a cognition-based construct), AC (i.e., an affect-based construct) and turnover (i.e., a behavior) is also consistent with Tajfel’s work on minimal group situation (e.g. [18–20]) and with the literature on intergroup relations (e.g. [22–24]). A strong parallel can be drawn with stereotypes, prejudice and discrimination where prejudice is the proximal cause of discrimination, while stereotypes are considered as the distal one. Furthermore, that is in line with early conception of attitudes and the well-established attitude-behavior sequence (e.g. [25]).

In line with this theoretical rationale, it is thus reasonable to assume that OI has an effect on actual turnover by enhancing AC. Preliminary empirical evidence has been reported by Marique and Stinglhamber [27] who showed the mediating effect of AC in the OI-turnover intentions relationship. However, to our knowledge, no research has addressed the mediating role of AC in the relationship between OI and actual turnover. As a consequence, we hypothesized that OI reduces turnover by strengthening AC.

*Hypothesis 3*: AC mediates the negative relationship between OI and voluntary employee turnover.

### Study 1: Directionality of the Relationship Between OI and AC

Study 1 was designed to examine Hypothesis 1, which holds that OI is an antecedent of AC. We used a cross-lagged panel design with two measurement times to assess the directionality of the relationship between these two constructs. Indeed, according to several authors (e.g. [30,31]), panel designs provide the strongest evidence of causal direction in field studies as compared to designs with simultaneous measurement of the variables.

### Method

#### Sample and procedure

One thousand seven hundred and twenty three employees from a Belgian Federal Public Service returned usable questionnaires at Time 1 (response rate = 25.47%) and 695 at Time 2 (response rate = 40.34%). Given the important dropout between Time 1 and Time 2, we tested the potential effects of subject attrition by following Goodman and Blum’s [62] recommendations. We first determined whether subject attrition led to non-random sampling. A logistic regression was conducted, using a dichotomous dependent variable which classified subjects as either stayers (i.e., those who responded to the two survey questionnaires) or leavers (i.e., those who did not respond to the second questionnaire). The independent variables included all the variables of interest, i.e. OI, AC and the two control variables (organizational tenure and level of function; see below). Results indicated the presence of non-random sampling since the probability of remaining in the sample was predictable from organizational tenure, level of function and OI (*B* = .01, *p* < .05; *B* = .12, *p* < .01 and *B* = -.19, *p* < .05, respectively) but not from AC (*B* = .03, *n*.*s*.).

As recommended by Goodman and Blum [62], we thus went on to assess the effects of non-random sampling on our data and, in particular, on the relationships among variables. Multiple regression analyses were performed to test the relationships among the independent variables measured at Time 1 (i.e., organizational tenure in the organization, level of function, and OI) and the dependent variable also measured at Time 1 (i.e., AC). A multiple regression model on the whole sample was compared to a model including only those who responded to both data collections. Results indicated that OI is a significant predictor in both models (β = .57, *p* < .001 and β = .59, *p* < .001, respectively), whereas organizational tenure and level of function were significant in one model and not in the other (organizational tenure: β = .01, *n*.*s*. and β = .06, *p* < .05, respectively; level of function: β = .04, *p* < .05 and β = .05, *n*.*s*., respectively). However, t tests showed no significant difference in regression coefficients between the two models (*t* = -.01, *n*.*s*., and *t* = -.17, *n*.*s*., for organizational tenure and level of function, respectively). As a whole, this evidence suggests that attrition is not a significant bias in this study.

Of the final sample, 59.3% were male. The average age of participants was 48.5 years (*SD* = 8.98) and average organizational tenure was 22.32 years (*SD* = 11.22). Like most of the public companies in Belgium, this company was divided into seven main levels of function, each level corresponding to a specific level of knowledge, skills and abilities. Three percent of the respondents belong to level D (i.e., the lowest level), 18.7% to level C, 34.5% to level B, 13.5% to level A1, 25% to level A2, 4.6% to level A3, and 0.6% to levels A4 and A5 (i.e., the highest levels). The authors who collected the data for this study work in a university in which no institutional review board (IRB) existed at the time the study was launched. Nevertheless, the study was carried out according to the "Ethical Principles of Psychologist and Code of Conduct" of the American Psychological Association [63]. Particularly, participants were informed of the aims of the research, participation was voluntary, there was no adverse consequence of declining or withdrawing from participation, and confidentiality was protected since responses were kept anonymous. The first questionnaire, containing the variables of interest as part of a larger survey, was given out in an email providing a link to the electronic survey. The same questionnaire was administered again 4 months later, following the same procedure. This time lag was chosen in agreement with the requirements of the partner organization. In both questionnaires, respondents were asked to provide a personal code allowing us to match their responses to each questionnaire.

#### Measures

Because the study was conducted in a French-speaking context, all measures were translated from English into French using the standard translation-back-translation procedure recommended by Brislin [64]. A 5-point Likert-type scale was used in order to measure respondents' level of agreement with each item (1 = *"Strongly disagree"*; 5 = *"Strongly agree"*).

***OI*.** The 6-item scale developed by Mael and Ashforth [3] was used to measure employees' OI (e.g. "When I talk about [organization name], I usually say 'we' rather than 'they'").

***AC*.** We used the revised 6-item scale of Meyer and his colleagues [65] to measure employees' AC (e.g. "I do not feel "emotionally attached" to [organization name]"). However, some authors have recently argued that there is an overlap between OI and AC at the measurement level (e.g. [12]). More precisely, one item of the Affective Commitment Scale (i.e., "I really feel as if [organization name]'s problems are my own") is considered to tap into the OI construct (e.g. [37,66]). Therefore, following Conway and Lance's [67] recommendation, we dropped this item in order to prevent artifactual inflation of the relationship between OI and AC.

***Control variable*.** Following Becker's [68] recommendations, only demographic variables displaying significant correlations with the dependent variables of our model were controlled for in the subsequent analyses. Organizational tenure and level of function were found to be significantly related to Time 2 OI (*r* = .10, *p* < .05 and *r* = .15, *p* < .001, respectively) and Time 2 AC (*r* = .10, *p* < .01 and *r* = .08, *p* < .05, respectively). We therefore decided to introduce organizational tenure and level of function as additional exogenous variables predicting Time 2 OI and Time 2 AC.

### Results

#### Discriminant validity

Using confirmatory factor analyses (Lisrel 8.8; [69]), we examined the distinctiveness between OI and AC at each measurement time. At both Time 1 and Time 2, a two-factor model was found to yield a good fit to the data (χ^2^(43) = 175.95; RMSEA = .07, *p* < .01; CFI = .98; NNFI = .97, and χ ^2^(43) = 187.40; RMSEA = .07, *p* < .001; CFI = .98; NNFI = .98, respectively) and the one-factor model displayed significant decrements in fit as compared with the two-factor model (Δχ ^2^(1) = 429.50, and Δχ ^2^(1) = 411.56, respectively), confirming the discriminant validity of OI and AC.

#### Relationships among variables

Descriptive statistics, reliability coefficients, and intercorrelations among variables are presented in Table 1. Of greatest interest, Time 1 OI was positively related to Time 2 AC.

**Table 1:** Study 1: Descriptive Statistics and Intercorrelations Among Variables.

Variable	*M*	*SD*	1	2	3	4	5	6
1. Organizational tenure	22.32	11.22	—		*			
2. Level of function	—	—	-.02	—				
3. Time 1 OI	3.02	.74	.10	.11**	(.84)			
4. Time 1 AC	3.02	.77	.12**	.11**	.60***	(.81)		
5. Time 2 OI	2.99	.76	.10*	.15***	.74***	.53***	(.87)	
6. Time 2 AC	3.05	.74	.10**	.08*	.56***	.74***	.61***	(.79)

*Note*. *N* = 695. α coefficients are reported on the diagonal. OI = organizational identification; AC = affective organizational commitment. Level of function was coded 1 for level D, 2 for level C, 3 for level B, 4 for level A1, 5 for level A2, 6 for level A3, and 7 for levels A4 and A5.

*p < .05.

**p < .01.

***p < .001.

#### Test of hypotheses

Using structural equation modeling (Lisrel 8.8; [69]), we assessed the relationship between Time 1 OI and the temporal change in AC, and between Time 1 AC and the temporal change in OI. Based on Finkel's [30] recommendations, variances between Time 2 OI and Time 2 AC were allowed to covary and the error covariances of identical items over time were also allowed to correlate.

**Fig 1:**
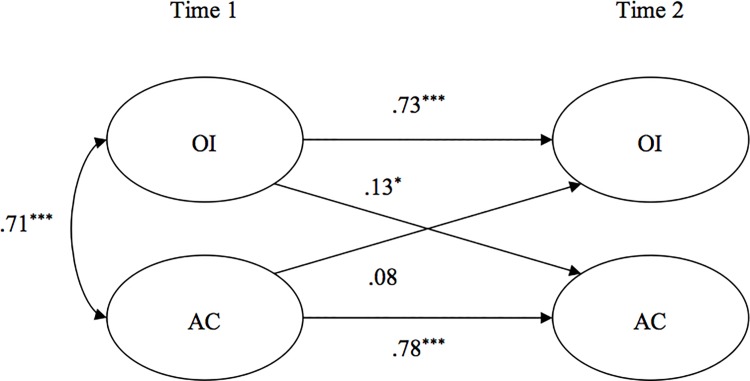
Study 1: Structural Equation Model of the Relationships Between Organizational Identification (OI) and Affective Organizational Commitment (AC) Over Time. *p < .05. **p < .01. ***p < .001.

Standardized parameter estimates for the cross-lagged model are displayed in Fig 1. For ease of presentation, only structural relationships are shown and the effects of the two control variables (i.e., organizational tenure and level of function) are described in the text. Organizational tenure was not significantly related to Time 2 OI and Time 2 AC (γ = .02, *n*.*s*. and γ = -.01, *n*.*s*., respectively). Level of function was significantly associated with Time 2 OI (γ = .06, *p* < .05) but not significantly related to Time 2 AC (γ = -.01, *n*.*s*.). As predicted, controlling for organizational tenure and level of function, OI was positively related to temporal change in AC (γ = .13, *p* < .05), while AC was not associated with temporal change in OI (γ = .08, *n*.*s*.). Moreover, the overall model yielded a good fit to the data (χ^2^(228) = 553.54; RMSEA = .05, *n*.*s*.; CFI = .99; NNFI = .98). In sum, consistent with Hypothesis 1, OI was found to be positively related to temporal change in AC, whereas the reverse was not true, providing evidence that OI leads to AC.

## Study 2: The Mediating Role of OI in the Relationship Between Favorable Work Experiences and AC

Study 2 was designed to assess Hypothesis 2 (a, b, and c) that holds that OI mediates the relationships between three favorable work experiences (i.e., perceived organizational support, leader-member exchange, and job autonomy) and AC.

### Method

#### Sample and procedure

The present sample originates from the same Belgian Federal Public Service as Study 1. More precisely, it comprised the 1723 employees who responded to the questionnaire at the first measurement time of Study 1. Fifty-four point three percent were male. The average age of participants was 47.85 years (*SD* = 9.33) and average organizational tenure was 21.79 years (*SD* = 11.53). On average, employees had worked with their supervisor for 5.37 years (*SD* = 5.11). Of this final sample, 5.7% of the respondents belong to level D, 20.1% to level C, 32.7% to level B, 14.7% to level A1, 21.6% to level A2, 4.2% to level A3, and 0.8% to levels A4 and A5.

#### Measures

Because the study was conducted in a French-speaking context, all measures were translated from English into French using the standard translation-back-translation procedure [64].

***Leader-member exchange*.** Employees were asked to report the quality of the exchange relationship between them and their supervisor using the 7-item scale of Graen and Uhl-Bien [70] (e.g. "Do you usually know how satisfied your supervisor is with what you do?"). Each item was associated with a specific 5-point Likert-type scale.

***Perceived organizational support*.** Employees' perception of organizational support was measured using the shorter 8-item version of the Survey of Perceived Organizational Support (SPOS; [36]) (e.g. "[Organization name] really cares about my well-being"). For this and the remaining measures, respondents were asked to rate their agreement with each item on a 5-point Likert-type scale (1 = *"Strongly disagree"*; 5 = *"Strongly agree"*).

***Job autonomy***. We used the 3-item scale developed by Fuller, Marler, and Hester [71] in order to measure employees' perceived job autonomy (e.g. "I have considerable opportunity for independence and freedom in how I do my job").

***OI and AC*.** We used the same scales as in Study 1.

***Control variable*.** Following Becker's [68] recommendations and consistently with the decision rules used in Study 1, we introduced organizational tenure and level of education as additional exogenous variables predicting OI. Organizational tenure, level of education, and level of function were also introduced as additional exogenous variables predicting AC.

### Results

#### Discriminant validity

The discriminant validity between perceived organizational support, leader-member exchange, job autonomy, OI, and AC was assessed through the comparison of nested measurement models (Lisrel 8.8; [69]). Results indicated that the hypothesized five-factor model fitted the data well (χ^2^(367) = 2057.10; RMSEA = .05, *n*.*s*.; CFI = .97; NNFI = .97) and was significantly superior to all more constrained models, obtained through the combination of constructs two by two (e.g. model combining OI and AC: χ^2^(371) = 3713.21; RMSEA = .07, *p* < .001; CFI = .96; NNFI = .95; Δχ^2^(4) = 1656.11, *p* < .001).

#### Relationships among variables

Descriptive statistics, reliability coefficients, and intercorrelations among variables are displayed in Table 2. Interestingly, perceived organizational support, leader-member exchange, and job autonomy were positively related to both OI and AC. Moreover, OI was positively related to AC.

**Table 2:** Study 2: Descriptive Statistics and Intercorrelations Among Variables.

Variable	*M*	*SD*	1	2	3	4	5	6	7	8
1. Organizational tenure	21.79	11.53	—							
2. Level of education	—	—	-.43***	—						
3. Level of function	—	—	-.03	.62***	—					
4. POS	2.31	0.64	-.01	-.07**	-.02	(.85)				
5. LMX	3.08	0.85	.07**	-.09***	-.08**	.30***	(.92)			
6. JA	3.66	0.93	.04	-.02	.07**	.26***	.35***	(.94)		
7. OI	3.07	0.75	.10***	-.08**	.04	.31***	.19***	.17***	(.84)	
8. AC	3.04	0.75	.06**	-.07**	.06*	.46***	.34***	.29***	.57***	(.81)

*Note*. *N* = 1723. POS = Perceived organizational support; LMX = Leader-member exchange; JA = Job autonomy; OI = organizational identification; AC = affective organizational commitment. α coefficients are reported on the diagonal. Level of education was coded 1 for primary education, 2 for lower secondary education, 3 for upper secondary education, 4 for bachelor, 5 for master, and 6 for Ph.D. Level of function was coded 1 for level D, 2 for level C, 3 for level B, 4 for level A1, 5 for level A2, 6 for level A3, and 7 for levels A4 and A5.

*p < .05.

**p < .01.

***p < .001.

#### Test of hypotheses

The hypothesized structural relationships among latent variables were assessed using the structural equation modeling approach (Lisrel 8.8; [69]). Fit indices for the hypothesized structural model and five alternative models are displayed in Table 3. As indicated in this table, the hypothesized model accurately explained the data. However, the fit of the alternative model 3, which adds direct paths between (a) perceived organizational support and AC, (b) leader-member exchange and AC, and (c) job autonomy and AC, was significantly better than the fit of more constrained models. We thus retained the alternative model 3 as the best depiction of the data.

**Table 3:** Study 2: Fit Indices for Structural Models.

Model	χ^2^	*df*	NNFI	CFI	RMSEA	Δχ^2^ (Δ*df*)	Model comparison
Hypothesized	2597.83	443	.96	.97	.05**	200.71(1)***	Hypothesized vs. Alternative 1
Alternative 1 (path added between POS and AC)	2397.12	442	.97	.97	.05	73.61(1) ***	Alternative 1 vs. Alternative 2
Alternative 2 (Alternative 1 + path added between LMX and AC)	2323.51	441	.97	.97	.05	6.51(1) ***	Alternative 2 vs. Alternative 3
Alternative 3 (Alternative 2 + path added between JA and AC)	2317.00	440	.97	.97	.05	—	—
Alternative 4 (Alternative 3 except that path between POS and OI = path between LMX and OI)	2336.51	441	.97	.97	.05	19.51(1) ***	Alternative 3 vs. Alternative 4
Alternative 5 (Alternative 3 except that path between POS and OI = path between JA and OI)	2343.10	441	.97	.97	.05	26.10(1) ***	Alternative 3 vs. Alternative 5
Alternative 6 (Alternative 3 except that path between LMX and OI = path between JA and OI)	2317.08	441	.97	.97	.05	0.08(1)	Alternative 3 vs. Alternative 6

*Note*. *N* = 1723. POS = perceived organizational support; LMX = leader-member exchange; JA = job autonomy; OI = organizational identification; AC = affective organizational commitment; NNFI = Non-Normed Fit Index; CFI = Comparative Fit Index; RMSEA = Root Mean Square Error of Approximation.

**p < .01.

***p < .001.

**Fig 2:**
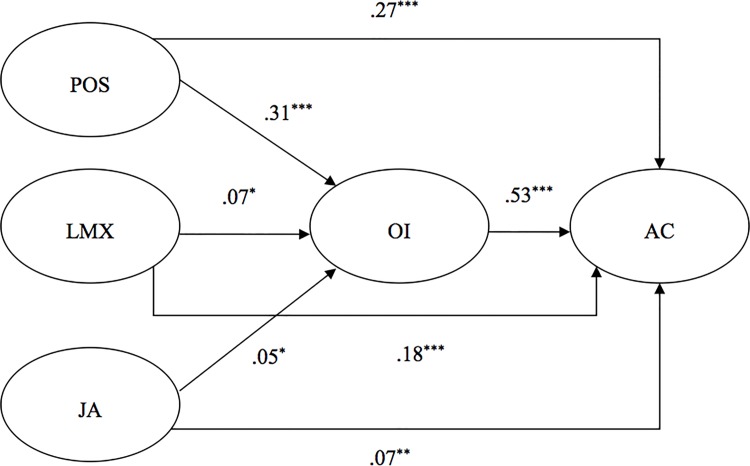
Study 2: Completely Standardized Path Coefficients for the Alternative Model 3. POS = perceived organizational support; LMX = leader-member exchange; JA = job autonomy; OI = organizational identification; AC = affective organizational commitment. For the sake of clarity, only structural relationships are shown. *p < .05. **p < .01. ***p < .001.

Standardized parameter estimates for the alternative model 3 are shown in Fig 2. For the sake of clarity, the effects of the three control variables are described in the text. Organizational tenure was found to be significantly related to OI (γ = .10, *p* < .001) while level of education did not significantly predict OI (γ = -.02, *n*.*s*.). Moreover, level of education and level of function were found to be significantly related to AC (γ = -.08, *p* < .05 and γ = .12, *p* < .001, respectively), whereas organizational tenure did not significantly predict AC (γ = -.02, *n*.*s*.). Controlling for these variables, the results showed that perceived organizational support, leader-member exchange, and job autonomy were positively associated with OI (γ = .31, *p* < .001; γ = .07, *p* < .05; and γ = .06, *p* < .05, respectively) which, in turn, has a significant and positive effect on AC (β = .53, *p* < .001). The indirect effects were tested with bootstrapping analyses using the PROCESS macro [72]. Results indicated that the indirect effects of perceived organizational support, leader-member exchange, and job autonomy on AC were all significant (indirect effect = .13, BCa 95% CI = [.11;. 17]; indirect effect = .04, BCa 95% CI = [.01;. 06]; and indirect effect = .02, BCa 95% CI = [.00;. 04], respectively), supporting Hypotheses 2a, 2b, and 2c. Perceived organizational support, leader-member exchange, and job autonomy were also found to be directly related to AC (γ = .27, *p* < .001; γ = .18, *p* < .001; and γ = .07, *p* < .01, respectively), indicating that OI only partially mediates these relationships.

Ancillary analyses were conducted in order to investigate whether perceived organizational support, leader-member exchange, and job autonomy equally contribute to explain the variance in OI. These exploratory analyses allowed us to assess the predictive power of each work experience in the development of OI and, subsequently, AC. This is particularly interesting given that the three work experiences included in this research do not emanate from the same source (i.e., the overall organization, the immediate supervisor, and the job itself). As indicated in Table 3, we ran three additional models in which path coefficients between the three work experiences and OI were constrained to be equal and chi-square difference tests were performed in order to compare these models with the alternative model 3 in which the path coefficients were allowed to differ. As evidenced in Table 3, the alternative model 3 provides a better fit than models in which the path between perceived organizational support and OI was constrained to be equal to (a) the path between leader-member exchange and OI (alternative model 4) and (b) the path between job autonomy and OI (alternative model 5), indicating that perceived organizational support is a stronger predictor of OI than leader-member exchange and job autonomy. Conversely, the alternative model 3 did not significantly differ from a model in which the path between leader-member exchange and OI was constrained to be equal to the path between job autonomy and OI (alternative model 6), indicating that leader-member exchange and job autonomy are equal predictors of OI.

## Study 3: The Mediating Role of AC in the Relationship Between OI and Employee Turnover

Study 3 was designed to examine longitudinally Hypothesis 3 that holds that AC mediates the relationship between OI and employee actual voluntary turnover. This longitudinal study was composed of three measurement times. As part of larger surveys, OI was measured at Time 1 whereas AC was assessed at Time 2, with a 5-month interval between the two data collections. Finally, actual turnover data was measured at Time 3, i.e. 1 year after Time 2.

### Method

#### Sample and procedure

This sample consisted of newcomers of the Belgian Army who were invited to fill in paper-and-pencil questionnaires during collective sessions. One thousand three hundred nineteen employees completed the survey at Time 1 (response rate = 88.82%) and 1012 at Time 2 (response rate = 76.72%). Of this final sample, 90.8% were male and average age was 20.87 years (*SD* = 3.55). Fifty percent were soldiers, 31.8% noncommissioned officers, and 18.2% officers. This study was approved by the Ethics Committee of the Psychology Department at the Université de Liège (Belgium). All participants gave their written informed consent. They were provided with full details regarding the aims of the study and the procedure, were totally free to join into the study, could quit the study at any time they wished, and were ensured that their responses would be kept confidential.

#### Measures

As the study was conducted in a French- and Dutch-speaking context, all measures were translated using the standard translation-back-translation procedure [64]. Respondents rated their agreement with each item using a 5-point Likert-type scale (1 = *"Strongly disagree"*; 5 = *"Strongly agree"*).

***OI and AC*.** We used the same scales as in Study 1 and Study 2.

***Turnover*.** Employee voluntary turnover data were obtained from organizational records. Stayers were coded 0 while leavers were coded 1.The turnover rate in the sample was 19.9%. This turnover rate is in line with previous studies showing that military turnover is usually higher than civilian turnover (e.g. [73]).

***Control variable*.** As in Study 1 and Study 2, demographic variables displaying a significant correlation with the mediator or the outcome variable of our model (i.e., gender, language, and level of function) were entered as covariates in the subsequent analyses.

### Results

#### Discriminant validity

Using confirmatory factor analyses (Lisrel 8.8; [69]), we examined the discriminant validity of Time 1 OI and Time 2 AC. Results indicated that a two-factor model fitted the data well (χ^2^(43) = 260.89; RMSEA = .07, *p* < .001; CFI = .96; NNFI = .95) and the one-factor model was found to display a significant decrement in fit as compared with the two-factor model (Δχ^2^(1) = 910.19), confirming the distinctiveness between Time 1 OI and Time 2 AC.

#### Relationships among variables

Descriptive statistics, reliability coefficients, and intercorrelations among variables are displayed in Table 4. As evidenced in this table, Time 1 OI was positively related to Time 2 AC and Time 2 AC was negatively related to actual turnover. However, Time 1 OI was not significantly related to actual turnover. According to several authors [74–78], finding a significant relationship between the independent variable and the dependent variable is not a necessary condition for mediation to occur and the indirect effect can be used as the criterion to conclude that mediation exists. We therefore decided not to consider the direct effect of OI on actual turnover as a prerequisite for mediation.

**Table 4:** Study 3: Descriptive Statistics and Intercorrelations Among Variables.

Variable	*M*	*SD*	1	2	3	4	5	6
1. Language	—	—	—					
2. Gender	—	—	-.02	—				
3. Level of function	—	—	.06	.16***	—			
4. Time 1 OI	3.38	0.62	.15***	-.05	.02	(.75)		
5. Time 2 AC	3.61	0.60	.15***	.01	-.06	.41***	(.77)	
6. Turnover	0.20	0.40	-.14***	-.06*	-.19***	-.03	-.11***	—

*Note*. *N* = 1012. OI = organizational identification; AC = affective organizational commitment. α coefficients are reported on the diagonal. Language was coded 1 for Dutch and 2 for French. Gender was coded 1 for male and 2 for female. Level of function was coded 1 for soldiers, 2 for noncommissioned officers, and 3 for officers. Turnover was coded 0 for stayers and 1 for leavers.

*p < .05.

**p < .01.

***p < .001.

#### Test of hypotheses

Because actual turnover was a dichotomous variable, logistic regression analysis was carried out using the bootstrapping technique [72,79] to examine whether Time 2 AC mediates the relationship between Time 1 OI and actual turnover. As evidenced in Table 5, controlling for language, gender, and level of function, the bootstrap results indicated that Time 1 OI is positively associated with Time 2 AC (*B* = .39, *p* < .001) which, in turn, has a negative relationship with actual turnover (*B* = -.56, *p* < .001). More importantly, the results of bootstrap analysis revealed that the 95% bias-corrected confidence interval for the size of the indirect effect does not include zero (indirect effect = -.22; BCa 95% CI = [-.36;-.10]), suggesting that Time 2 AC significantly mediated the relationship between Time 1 OI and actual turnover. As a whole, the results of this analysis support Hypothesis 3.

**Table 5:** Study 3: Logistic Regression Analysis.

Dependent variables	Time 2 AC		Time 3 Turnover	
	*B*	*SE*	*B*	*SE*
Constant	2.14***	.13	2.50	.72
Language	.12	.04	-.70***	.18
Gender	.08***	.06	-.43	.34
Level of function	-.06**	.02	-.73***	.13
Time 1 OI	.39***	.03	.19	.15
Time 2 AC			-.56***	.16

*Note*. *N* = 1012. Nagelkerke *R²* = .11. OI = organizational identification; AC = affective organizational commitment. Language was coded 1 for Dutch and 2 for French. Gender was coded 1 for male and 2 for female. Level of function was coded 1 for soldiers, 2 for noncommissioned officers, and 3 for officers. Turnover was coded 0 for stayers and 1 for leavers.

*p < .05.

**p < .01.

***p < .001.

## Discussion

To the best of our knowledge, our research is the first to test across three studies an integrated model of the employer-employee relationships including both OI and AC, and their common antecedents and consequences. More precisely, using a cross-lagged panel design, Study 1 found that OI leads to AC while the reverse cannot be stated. Study 2 provided evidence that OI mediated positive associations of perceived organizational support, leader-member exchange, and job autonomy with AC. Study 3 reported that AC mediates the negative relationship between OI and subsequent voluntary turnover. Taken together, the findings suggest that favorable work experiences operate via OI to increase AC, which, in turn, is negatively associated with employee turnover.

The positive relationship found between OI and temporal change in AC in Study 1 indicates that OI leads to AC. As such, this study provides a stronger indication of the direction of causality between OI and AC than did prior studies that assessed OI and AC simultaneously and on only one occasion. This finding is consistent with the dominant view in the organizational literature that OI plays a role in AC's development (e.g. [8,15]). It is also in line with previous literature in social psychology (particularly, Tajfel’s work on minimal group situation; e.g. [18–20]) by demonstrating in real organizational settings that the cognitive awareness of the organizational membership is the first step in the development of an emotional relationship with the organization and its subsequent behaviors.

Overall, our findings provide thus evidence that the cognitive dimension of the employee-employer relationship (i.e., OI) precedes the affective dimension of this relationship (i.e., AC). Nevertheless, we should note that some authors pointed out the potential overlap between the measures we used to assess OI and AC (e.g. [12,17]). These scholars argued that the Mael and Ashforth's [3] scale neglected the cognitive element of identification while its authors [9 p21] argued that OI is a "perceptual cognitive construct that is not necessarily associated with any specific behaviors or affective states" (e.g. [80,81]). Some authors even stated that Mael and Ashforth's [3] items have an affective connotation (e.g. [80]). As explained above, we tried to remedy part of this problem by removing one item of the Affective Commitment Scale which was argued to tap into the OI construct (e.g. [37,66]). This allowed us to prevent an artifactual inflation of the relationship between OI and AC, by increasing the discriminant validity of the two measures [66,67]. Furthermore, another criticism to the OI scale [3] was formulated by Abrams and de Moura who stated that this scale is "predominantly concerned with public expressions of identification rather than its subjective meaning" [82 p137]. Taken together, these arguments raise the question of whether this is the cognitive aspect of identification which predicts AC, or something more encompassing such as a multidimensional identification or the public expression of identification. Future research aiming at developing new OI and AC scales should probably take into consideration this overlap, and propose measures that are more distinct from one another and in line with their theoretical constructs.

Above and beyond the causal relationship between OI and AC, our research also examined the relationship between these two constructs and three of their common antecedents. While the social exchange theory is often mentioned to explain and understand the development of commitment in the workplace [35,83], OI has been found to mediate the relationships between perceived organizational support, leader-member exchange, job autonomy and AC in Study 2. These results indicate that social identity processes may provide a new insight into the work experiences-AC relationship. These results extend Marique et al.'s [29] findings that showed the mediating role of OI in the perceived organizational support-AC relationship. They suggest that by providing these favorable work experiences, the employer increases organizational attractiveness that in turn fulfills important socioemotional needs among employees (e.g. [37]). Individuals tend to identify with organizations that are perceived to have positive characteristics because membership of such organizations enhances self-esteem and meets the need for self-enhancement [55,84]. Providing its personnel with favorable work experiences contributes to the positive attributes of an organization that make identification with this organization more likely.

Interestingly, our ancillary analyses have shown that, among the three work experiences included in our research, perceived organizational support is clearly the most important predictor of OI and subsequent AC. This finding indicates that work experiences provided by the organization have more weight in the development of OI and AC than work experiences emanating from other sources (i.e., an agent of this organization such as the supervisor, or the job provided by this organization). These results are in line with the target similarity model [85] that proposes that the relationship between two concepts is stronger when they refer to similar targets rather than different ones. Because perceived organizational support, OI, and AC all refer to the organization as a whole, they are thus likely to be more highly related.

The important effect of perceived organizational support in comparison with that of leader-member exchange and job autonomy might also be explained by the fact that prior studies have shown leader-member exchange and job autonomy to be antecedents of perceived organizational support. Indeed, numerous studies have reported a positive and significant association between leader-member exchange and perceived organizational support (e.g. [50,86]). Furthermore, Eisenberger and his colleagues [87] have recently examined the direction of causality between these two constructs. Through a panel design, they showed that leader-member exchange was an antecedent of perceived organizational support while the reverse was not true. In the same vein, job autonomy has been reported to be an important predictor of perceived organizational support to the extent that job autonomy indicates the organization's trust in employees [88]. Because the supervisor and the job are both nested within the organization, favorable treatment from these sources would contribute to a more general feeling of favorable treatment from the organization as a whole (i.e., perceived organizational support).

Finally, the present research studied the relationship between OI and AC and one of their common outcomes, i.e. employee actual voluntary turnover. Using a longitudinal design, we found in Study 3 that employees who identify strongly with their organization show an increased emotional attachment to this organization and this, in turn, leads to decreased turnover. More precisely, our results indicated that OI has no direct effect on employee turnover and that this relationship is totally mediated by AC. From a theoretical point of view, results of this study demonstrate the proximal role of AC in the prediction of turnover as compared to OI. This finding is consistent with the literature showing that organizational commitment is among the best predictors of employee turnover (e.g. [89,90]). This result is also in line with Ashforth et al. [17] who argued that OI is a "root construct" and that outcomes usually associated with OI are often quite distal. Even though the same hedonistic approach–avoidance mechanism probably underlies both the OI-turnover and the AC-turnover relationship [59], our results interestingly showed that OI and AC do not play the same role in the prediction process of turnover. OI seems to precede AC in the prediction of turnover, so that mediation effects occur among the variables pertaining to the same category of antecedents as described in Maetz and Griffeth’s [59] framework.

### Limitations and Future Research

This research has several limitations which also suggest interesting perspectives for future research. First, in Study 1, although we controlled for organizational tenure and level of function, we cannot rule out the possibility that the observed association between OI at Time 1 and AC at Time 2 was due to some omitted variable [30]. More importantly, the results of Study 1 certainly need replication with other samples but also with other intervals. While the present research examined OI and AC over a 4-month interval, it would be worthwhile investigating whether the effect of OI on AC is maintained over a longer interval. More importantly, we cannot assume that the duration required for OI to influence AC is the same as that required for AC to influence OI [91]. Future research might examine the possibility of such a longer-term relationship by assessing OI and AC several times and using longer intervals between measurement occasions.

More importantly, Study 1 and 2 were conducted among a population of workers having a relatively long organizational tenure, whereas Study 3 was carried out among a population of newcomers. According to the literature on newcomers (e.g. [92]), these employees experience a high level of uncertainty and stress due to a “reality shock” upon their organizational entry linked to their news roles, tasks and norms. “Newcomers are unlikely to begin their jobs with a fully developed sense of identification with their new organization” [58 p47] and develop their identity through a series of phases over time [93]. Therefore, it may be possible that the causal relationship between OI and AC is more prone to evolve and change over time among a population of newcomers.

In the same vein, prior research on organizational change found that employees’ perceptions of uncertainty in their work environment lead to either reinforce [94] or reduce their OI (e.g. [95]). According to Amiot and her colleagues, “because mergers require employees to relinquish their premerger organizational identification and to identify with the new merged organization, this social change not only involves adopting new work procedures and developing productive relationships with members of a previously separate organization, it also means redefining an important aspect of one’s work identity” [93 p364]. Similarly, research has consistently showed that AC may be profoundly impacted as a result of important external events such as a merger or a layoff (e.g. [34,96]. In light of this evidence, it is possible that the findings of Study 1 and 2 may not be generalizable to early stages of socialization and, more generally, that the conclusions of our research cannot be extended to situations of organizational changes. Clearly, future research would benefit to examine whether our research results are replicated among other populations of workers and in other organizational contexts.

Second, future research should also replicate our second study using, for example, a longitudinal design. Due to its cross-sectional design and the use of self-reported measures, the results of this study may indeed have been exposed to the common method bias. However, the use of self-reported measures seems the most accurate way in regard to the main purpose of our research, which was to examine employees’ perceptions, cognitions and attitudes. Additionally, we were able to partially address the concern over method bias by assuring participants of the confidentiality of their responses and asking them to answer questions as honestly as possible [97]. Finally, the problem of common method variance was partially addressed since results of confirmatory factor analyses revealed that a single-factor model showed a poor fit to the data (i.e., Harman’s single-factor test; [97]). As a whole, this evidence suggests that common method variance is probably not a pervasive problem in this study [97,98].

Third, our second study showed that the relationships between perceived organizational support, leader-member exchange and job autonomy on the one hand, and AC on the other hand, were partially mediated by OI. Providing its personnel with favorable work experiences contributes to the positive attributes of an organization that make identification with this organization more likely. Future research is however needed to further clarify this underlying process. Some authors have proposed another route that might explain the positive relationship between work experiences and OI. The group engagement model [99] suggests that OI is based not only on individuals' evaluation of the status of the organization, but also on individuals' evaluation of their own status within the organization. This latter evaluation refers to the extent to which individuals feel themselves to be members in good standing within the organization [100]. Yet, one may reasonably assume that being the 'target' of favorable work experiences may inflate this perception. Future studies on work experiences-OI relationships should thus examine more precisely the "avenues by which a membership group may provide individuals with a positive view of themselves" [100 p817].

Fourth, the present research focused on the relationship between OI and the affective dimension of organizational commitment because of their apparent conceptual similarity and the frequent confusion between the two concepts in the literature. However, to the best of our knowledge, no research has deeply investigated the impact of OI on the other two dimensions of organizational commitment, i.e. normative and continuance commitment. This is quite surprising given the potential influence that OI could have on these two dimensions of commitment. Indeed, OI is very likely to contribute to the development of normative commitment to the extent that individuals could develop a sense of obligation toward the organization in response to the benefits they derive from their organizational membership (e.g. high self-esteem). Furthermore, Riketta and van Dick argued that normative commitment could be considered as a consequence of OI to the extent that OI might lead to "the internalization of organizational reciprocity norms" [66 p74]. The same is true for continuance commitment or, at least, its 'high sacrifices' dimension. Indeed, leaving the organization could be perceived as a cost for individuals to the extent that the loss of their organizational membership would mean losing a part of their self-concept. Therefore, we think that future research should investigate how OI influences normative and continuance commitment.

### Practical implications

The present research suggests that organizations should strengthen employees' perceptions of organizational support, promote high-quality relationships between employees and their supervisor, and enhance employees' job autonomy in order to increase employees' identification and emotional attachment with the organization, ultimately decreasing turnover. Overall, our results do indicate that perceptions of organizational support, high-quality relationships with the supervisor, and job autonomy are powerful levers for organizations wishing to reduce the voluntary turnover among personnel through better employee-employer relationships.

Such positive work experiences can be achieved via diverse human resources practices. Indeed, according to Eisenberger and Stinglhamber [37], human resources practices such as maintaining open channels of communication, providing employees with necessary resources (i.e., equipment, training, information, or supplies) or with more job security should enhance their perceptions of organizational support. Moreover, a workgroup climate that emphasizes consensus in decision-making, cooperation, warmth, and friendliness should help to build high-quality relationships between employees and their supervisor [101]. More generally, organizations should sensitize managers to the relationships they develop with subordinates and encourage them to form high-quality relationships with all of the latter [102]. Finally, organizations should provide their employees with more job autonomy by giving them more independence and freedom to set their own schedules and choose how to do their work.

In sum, the present research indicates that employees' identification with the organization plays an important role in the relationship between their work experiences and the emotional attachment they develop toward the organization. Furthermore, our findings indicate that employees' voluntary turnover is influenced by their identification with the organization through its impact on employees' attachment toward the organization.

## Supporting Information

S1 Dataset Raw Data of Study 1. (SAV)

S2 Dataset Raw Data of Study 2. (SAV)

S3 Dataset Raw Data of Study 3. (SAV)
